# Energy Level-Based Abnormal Crowd Behavior Detection

**DOI:** 10.3390/s18020423

**Published:** 2018-02-01

**Authors:** Xuguang Zhang, Qian Zhang, Shuo Hu, Chunsheng Guo, Hui Yu

**Affiliations:** 1 The Institute of Electrical Engineering, YanShan University, Qinhuangdao 066004, China; zhangxg@hdu.edu.cn (X.Z.); zhangqian1@nuctech.com (Q.Z.); hus@ysu.edu.cn (S.H.); 2 School of Communication Engineering, Hangzhou Dianzi University, Hangzhou 310018, China; gcs@hdu.edu.cn; 3 LargeV Instrument Corporation Limited, Beijing 100084, China; 4 School of Creative Technologies, University of Portsmouth, Portsmouth PO1 2DJ, UK

**Keywords:** crowd abnormal detection, energy-level, flow field visualization, co-occurrence matrix

## Abstract

The change of crowd energy is a fundamental measurement for describing a crowd behavior. In this paper, we present a crowd abnormal detection method based on the change of energy-level distribution. The method can not only reduce the camera perspective effect, but also detect crowd abnormal behavior in time. Pixels in the image are treated as particles, and the optical flow method is adopted to extract the velocities of particles. The qualities of different particles are distributed as different value according to the distance between the particle and the camera to reduce the camera perspective effect. Then a crowd motion segmentation method based on flow field texture representation is utilized to extract the motion foreground, and a linear interpolation calculation is applied to pedestrian’s foreground area to determine their distance to the camera. This contributes to the calculation of the particle qualities in different locations. Finally, the crowd behavior is analyzed according to the change of the consistency, entropy and contrast of the three descriptors for co-occurrence matrix. By calculating a threshold, the timestamp when the crowd abnormal happens is determined. In this paper, multiple sets of videos from three different scenes in UMN dataset are employed in the experiment. The results show that the proposed method is effective in characterizing anomalies in videos.

## 1. Introduction

Abnormal crowd analysis [1,2,3] has become a popular research topic in computer vision. Currently, there are two main approaches in modeling the crowds. (1) The microscopic approach, which treats the crowd as a collection of individuals. In this approach, to identify the crowd behavior, each individual is detected and his movement is tracked [4,5,6,7,8,9]. Such a kind of method is suitable for dealing with a small-scale crowd. However, it is difficult to accurately detect and track all the individuals in a dense crowd due to the occlusions among some individuals; (2) The macroscopic approach, which considers a large-scale crowd as a single entity [10]. It treats each image pixel as a particle, and models the features of the particles to further identify the crowd behavior [11,12,13,14,15,16]. Many approaches based on the global analysis of a crowd have been developed. For example, in [17], a kinetic energy model of the crowd is built to detect the abnormal behavior. In [18], the authors use Social Force Model to calculate the intensity of force between the particle and the surrounding space to describe the pedestrian behavior. In [19], a gradient model based on space and time are proposed to detect partial abnormal crowd behavior. These types of methods do not require detection and tracking of the individual, which can reduce the final detection errors effectively. Therefore, more methods utilize the motion particles instead of the pedestrians to analyze the crowd behavior. 

In this paper, we develop a novel model to find features that can describe the normal and abnormal state of the crowd. The change of energy-level distribution information is applied to describe the crowd behavior. We adopt the kinetic theory to describe the energy of the particles. In the kinetic model, quality is an important attribution of a particle. For a particle in an image, it represents a tiny part of an object in real scene. Therefore, if the location of a pedestrian is far away from the camera, the size of this pedestrian will be smaller in this image, the particle correspond to a larger part of this pedestrian. On the contrary, if the location of the pedestrian is near by the camera, the particle means a smaller part of this pedestrian. Thus, the camera perspective effect should be taken into account. In this paper, the qualities of different particles are distributed as different value according to the distance between the particle and the camera to reduce the camera perspective effect. The method not only can reduce the camera perspective effect, but also can detect abnormal crowd behavior in time. The rest of the paper is organized as follows: Section 2 introduces the moving particles’ velocity and quality extraction method, and proposes a kinetic energy model; Section 3 discusses the method for quantitative grading for kinetics. The description will also be presented for the energy-level distribution of the moving particles using co-occurrence matrix; Section 4 presents the experimental results of different video clips and comparisons with other methods. Section 5 summarizes the paper.

## 2. Overview of the Method 

**Figure 1:**
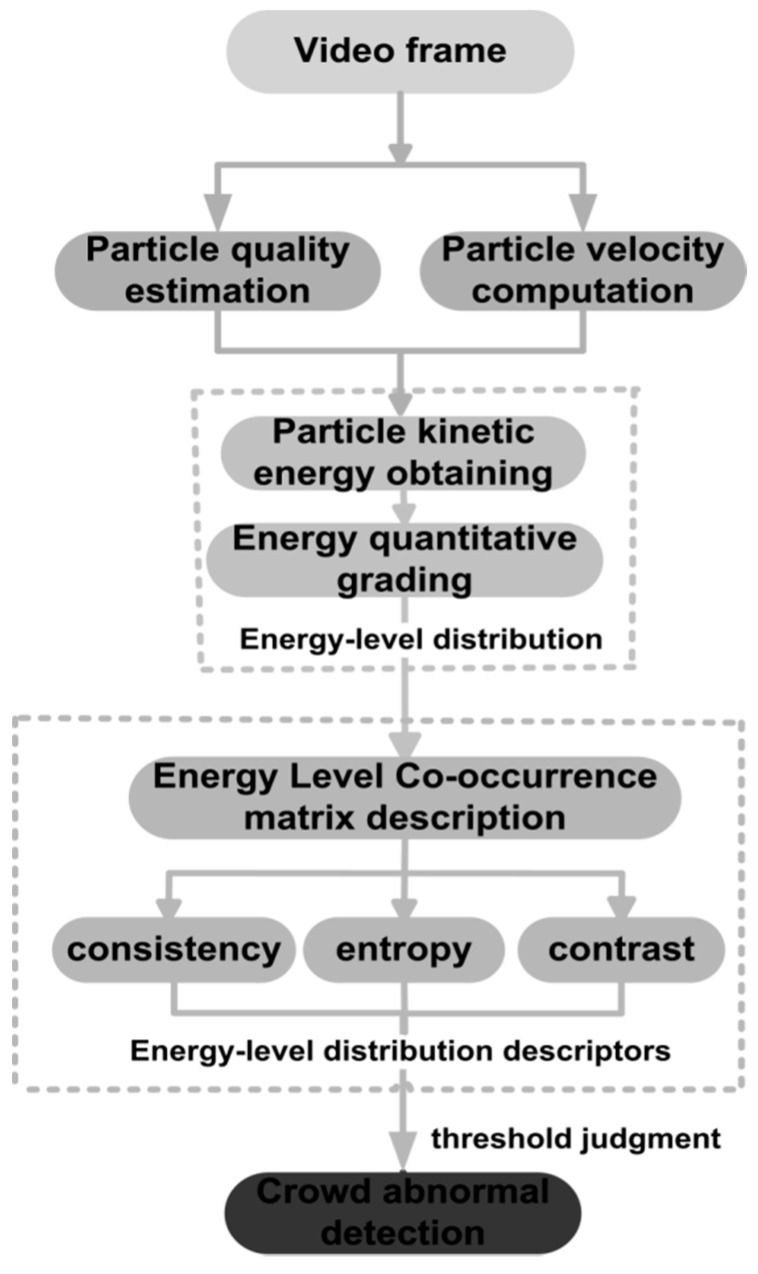
The framework of the proposed method.

This paper proposes a novel crowd anomaly detection method based on the change of energy-level distribution. Firstly, each pixel of the image is considered as a particle, and Horn–Strunck optical flow method [20] is adopted to extract the particle’s velocity. Then, two reference individuals that respectively locate further and closer to the camera are selected. Their foregrounds are extracted with the flow field visualization in the image [21]. Traditional foreground detection methods, such as background subtraction and Gaussian mixture model, are limited by phenomena of the inside holes once the appearances of target of interest and its background are similar. The reason for this drawback is that these methods only use the intensity information of every isolated pixel in the current image frame. However, the flow field visualization based method not only considers the information of a pixel in the image, but also the pixels on the same streamline, which can eliminate the error effectively. Next, the linear interpolation is applied for the two reference persons’ foreground area, and then the different qualities of particles that have different distance from the camera are calculated, which will weaken the influence of the camera perspective effect. Then according to the velocity and quality information of particle, a particle kinetic model is built, and the kinetic energy of each motion particle in the video is calculated. Secondly, the particle kinetic is analyzed for quantitatively grading, and the energy-level distribution of an image is obtained. In a normal state, the particles are usually in a low level. Therefore, the energy-level distribution is relatively single. When pedestrians become abnormal, some particles transit to the different high energy-level, which leads to a relatively more confused energy-level distribution. Finally, the distribution of particle’s energy-level is described with three co-occurrence matrix descriptors of uniformity, entropy and contrast. Whether the crowd abnormal behavior occurs will be determined by comparing the value of the descriptors with their corresponding threshold. When all three descriptors are determined as abnormal, an alarm prompt will be raised. We test multiple sets of videos in this paper, and the results show that our method can identify the crowd abnormal behavior effectively. The framework of the proposed method is shown in Figure 1.

## 3. Kinetic Energy Model 

### 3.1. Particle Velocity Computation 

**Figure 2:**
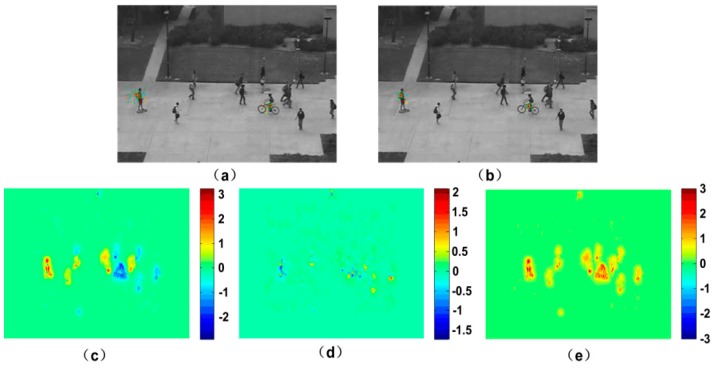
Optical flow of two frames: (**a**) Frame 41; (**b**) Frame 42; (**c**) horizontal optical flow; (**d**) vertical optical flow; (**e**) total optical flow.

We consider each pixel of the image as a moving particle, and use the Horn–Schunck (HS) optical flow method to extract the velocity of the particles. The HS method is a differential calculation method using optical flow. It assumes that the change of particles’ optical flow is smooth. In other words, the motion of the pixels not only satisfies the optical flow constraint, but also the global smoothness constraint. The specific operation for video sequence is as following: first, the point of coordinate (*x*, *y*) in the current frame is extracted, and the corresponding intensity I (*x*, *y*, *t*) at time *t* are obtained. Then the optical flow vector between the current and the next frame in horizontal and vertical direction respectively can be calculated according to (1):

(1) $$\begin{array}{c} u^{n+1}={\bar{u}}^{n}-I_{x}\frac{I_{x}{\bar{u}}^{n}+I_{y}{\bar{v}}^{n}+I_{t}}{\alpha^{2}+I_{x}^{2}+I_{y}^{2}}\\ v^{n+1}={\bar{v}}^{n}-I_{y}\frac{I_{x}{\bar{u}}^{n}+I_{y}{\bar{v}}^{n}+I_{t}}{\alpha^{2}+I_{x}^{2}+I_{y}^{2}} \end{array}$$

where *n* is the number of the iterations. $I_{x}=\partial I/\partial x$, $I_{y}=\partial I/\partial y$ and $I_{t}=\partial I/\partial t$ represent the differential of the pixel intensity in the direction of *x*, *y* and *t* respectively. u=∂x/∂t, v=∂y/∂t is the velocity in the *x* and *y* direction, and α is the parameter to control the smoothness. *u*_0_ and *v*_0_ are the initial estimates of optical flow field, and can be assigned zero generally. According to this method, we can calculate the moving particle’s velocity in the horizontal and vertical direction respectively. Figure 2 is the optical flow result obtained with HS method for two consecutive video frames in which the pedestrian moving in a road horizontally. The total optical flow includes the superposition of horizontal and vertical optical flow.

### 3.2. Particle Quality Estimation 

The movement of pedestrians is described using the motion of the particles. However, due to the camera perspective effect, there is a different number of particles, which causes different camera distances for a person. In this paper, different numbers of particles are assigned to different situations to reduce this deviation. To this end, an effective method that extracts the foreground of moving target is used to remove the pedestrians regions. Then, the area interpolation method is employed to calculate the qualities of different particles. The further the distance from camera is, the larger quantity of the particle will be assigned. 

#### 3.2.1. Foreground Extraction

The method in [21] is adopted in this paper to extract the foreground. This method uses the velocity vector and white noise to make the foreground of moving targets visualized by LIC (line integral convolution) [22,23]. Then, it calculates the image gray entropy [24] and uses threshold segmentation method [25] to get the foreground of the image.

**Figure 3:**
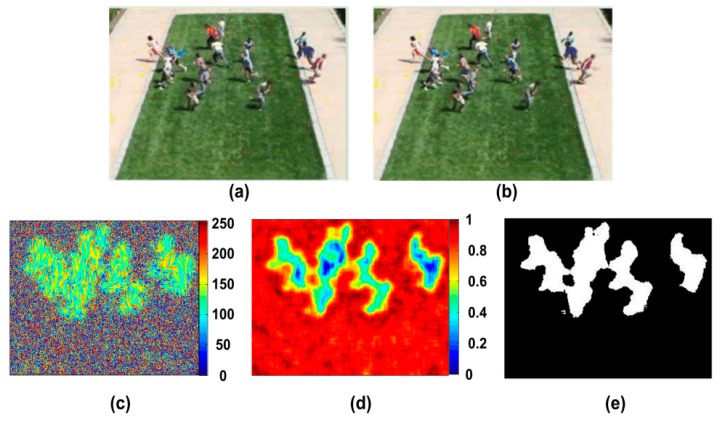
The result of foreground extraction, (**a**,**b**) are two consecutive frames of a crowd video; (**c**) is the result of LIC; (**d**) is the entropy image; (**e**) is the result of segmentation by Otsu method.

**Figure 4:**
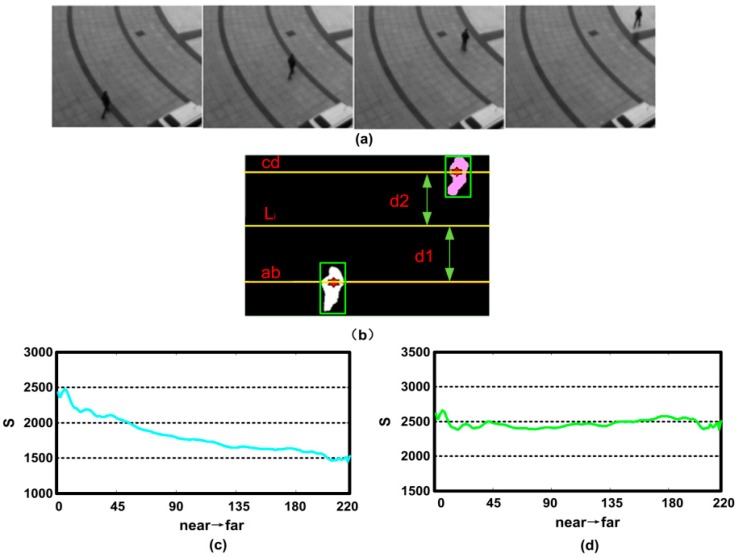
(**a**) Sample frames; (**b**) foreground of extraction and marked result; (**c**) area change curve of pedestrian; (**d**) area change curve of pedestrian after the improvement.

The white noise, which is the random distribution of black and white image, is firstly acquired, and then the velocity vector of the image is calculated based on optical flow. In the experiment of this paper, two consecutive frames are chosen from the video in which people run on the grass in an outdoor scene. The experiment result is shown in Figure 3c. The pedestrian movement is represented as a gray-scale image. We can see that the distributions of grey values for the motion and background regions are different. The texture of background region is rougher than the one of motion region. The gray entropy [26] is utilized to characterize the image texture information, which can be define as:

(2) $$H\left(z\right)=-\overset{L-1}{\underset{i=0}{\sum }}p\left(z_{i}\right)\log_{2}p\left(z_{i}\right)$$

where *p* is a probability distribution, and *L* is the number of different gray level. Entropy is the variable where the higher value represents the high disorder. Therefore, for a video with moving crowd, the entropies of the motion regions are low, while high for the background regions. It can be seen in Figure 3d, where the entropies are calculated for every 7 × 7 pixels region. Accordingly, a threshold can be determined to segment the moving crowd and background by Otsu method, which is shown in Figure 4e as the foreground extraction result. For the details of the process, please refer to the reference [21].

#### 3.2.2. Quality Estimation

In general, pedestrian looks small in the video surveillance. Therefore, the tiny difference between pedestrians caused by the different sizes and heights (such as man and women) will not be considered. We select two reference persons that have further and the nearer distance to the camera using the rectangular, then extract their foregrounds with the above method. Assume that the area of a person is composed of all the pixels of its foreground. This area of reference person is defined as *S*:

(3) $$S=\sum_{i=1}^{h}\sum_{j=1}^{w}M_{\mathrm{ij}}$$

where *h* and *w* denote the width and height of the rectangular respectively. $M_{\mathrm{ij}}\in \left\{0,1\right\}$, denotes the foreground by the value of 1 and denotes the background by 0. Figure 4a shows the sample frames of the scene where a pedestrian moves away from the camera gradually. We treat the pedestrian in the 1st and 220th frame as the two reference persons that have nearer and further distance to the camera respectively. Then, their foregrounds are extracted to find the center of mass of the references, which are marked with the red “*” in Figure 4b, Two horizontal lines are then drawn passing those two points. The reference line that close to the camera is called $\bar{\mathrm{ab}}$, and the one far from the camera is called $\bar{\mathrm{cd}}$. The labeling results are shown in Figure 4b. If a pedestrian moves from $\bar{\mathrm{ab}}$ to $\bar{\mathrm{cd}}$, the change of this person’s area in the scene is described as follows.

(4) $$k=\frac{S_{\mathrm{cd}}}{S_{\mathrm{ab}}}$$

Assume that the quality of the pixels on the line $\bar{\mathrm{ab}}$ is $m_{\mathrm{ab}}=1$, and the one on the line $\bar{\mathrm{cd}}$ is $m_{\mathrm{cd}}=1/k$. The quality of pixels can be obtained on the line of $L_{i}\left(0\leq i\leq H,\;\mathrm{where}\;H\;\mathrm{is}\;\mathrm{the}\;\mathrm{height}\;\mathrm{of}\;\mathrm{the}\;\mathrm{image}\right)$. It has the distance from $\bar{\mathrm{ab}}$ as $d_{1}$ and the distance from $\bar{\mathrm{cd}}$ as $d_{2}$ according to a linear interpolation method:

(5) $$m_{i}=\frac{m_{\mathrm{ab}}+\frac{d_{1}}{d_{2}}m_{\mathrm{cd}}}{1+\frac{d_{1}}{d_{2}}}=\frac{d_{2}\times k+d_{1}}{k\times \left(d_{1}+d_{2}\right)}$$

The particles have a same quality at the same ordinate line. The quality of particles with coordinates (i,j) is $m_{\mathrm{ij}}=m_{i}\left(0\leq i\leq H,\;0\leq j\leq W,\;W\;\mathrm{is}\;\mathrm{the}\;\mathrm{width}\;\mathrm{of}\;\mathrm{the}\;\mathrm{image}\right).$ In order to verify the feasibility of our method, we make a statistical analysis on the pedestrian area in the above scene. Because there is only one moving object in the scene, we redefine the area of reference person as following:

(6) $$S_{\mathrm{improve}}=\sum_{i=1}^{H}\sum_{j=1}^{W}m_{\mathrm{ij}}M_{\mathrm{ij}}\;$$

The quality of each particle is initialized as 1 making *m_ij_* = 1. Figure 4c shows the area change curve, and we can see that the person area reduces while the distance to the camera increases. Secondly, the area of reference person is acquired by Equation (6). It can be seen that the variance of the curve is becoming relatively flat in Figure 4d, which proves that our method is feasible. 

### 3.3. Particle Kinetic Energy Model 

According to the velocity and quality information of particle, a particle kinetic model can be established. The kinetic energy of particle with the coordinate of (*i*, *j*) is defined as following:

(7) $$E_{k}\left(i,j\right)=\frac{1}{2}m_{\mathrm{ij}}{\left(\mathrm{uv}\right)}_{\mathrm{ij}}^{2}$$

where $m_{\mathrm{ij}}$ is the quality of the particle with the coordinate of (*i*, *j*). ${\left(\mathrm{uv}\right)}_{\mathrm{ij}}$ is the resultant velocity in horizontal or vertical direction. It is defined as following:

(8) $${\left(uv\right)}_{\mathrm{ij}}=\sqrt{u_{{\mathrm{ij}}^{2}}+v_{{\mathrm{ij}}^{2}}}$$

## 4. Energy-Level Distribution of Crowd 

In this section, we carry on the quantitative grading to the kinetic energy, and retrieve the energy-level distribution of particles. Then the energy-level distribution is described by the co-occurrence matrix. The descriptors of co-occurrence matrix are adopted to describe the crowd state.

### 4.1. Energy Grading of Particles 

Modern quantum physics indicates that an outer electrons’ state is discontinuous. Therefore, the corresponding energy is also discontinuous. This energy value is called energy-level. Under normal condition, the atoms are in the lowest energy states, which are called the ground states. When atoms are excited by energy, their outer electrons transit to the different energy states, which are called, excited states.

In order to describe the energy-level distribution of a crowd, the movement of motion particles in an image are assumed as electronic movements. The kinetic energy of particles is treat as the whole energy of it. In normal state, people walk in low speeds, where the motion particle energies are low. Thus, most particles are in ground state. In abnormal state, people start running and particle energies rise suddenly, which make the some of the motion particles transit to higher levels. According to the hydrogen atom energy level formula, we can acquire a particle’s energy level by

(9) $$l=\sqrt{E_{\mathrm{excited}}/E_{\mathrm{ground}}}$$

where $E_{\mathrm{excited}}$ is the kinetic energy of the excited state, and $E_{\mathrm{ground}}$ is the kinetic energy of the ground state. We treat the average kinetic energies of motion particles in normal state as the ground state energies. In addition, *l* will always be rounded down to make sure that the energy level of particles is an integer.

### 4.2. The Description of Energy-Level Co-Occurrence Matrix 

**Figure 5:**
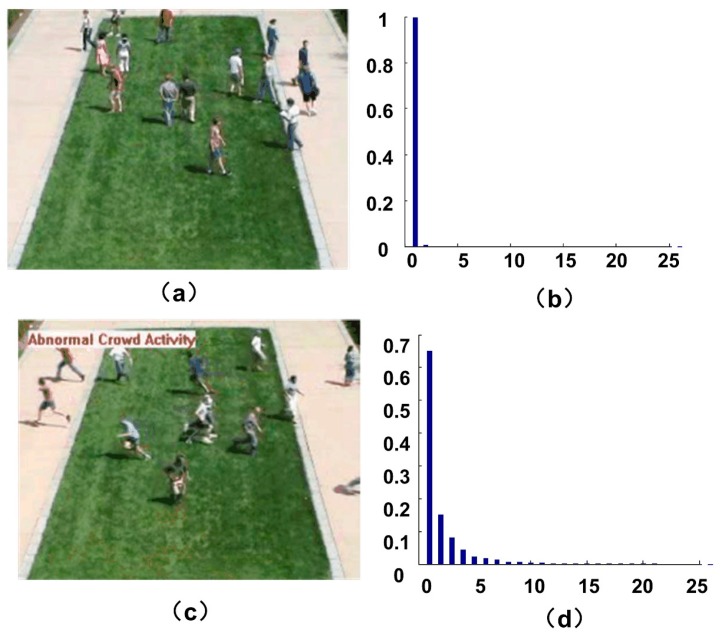
Energy-level distribution in normal and abnormal scene. (**a**,**c**) are two frames of normal and abnormal scenes; (**b**,**d**) are the energy-level distributions respectively.

**Figure 6:**
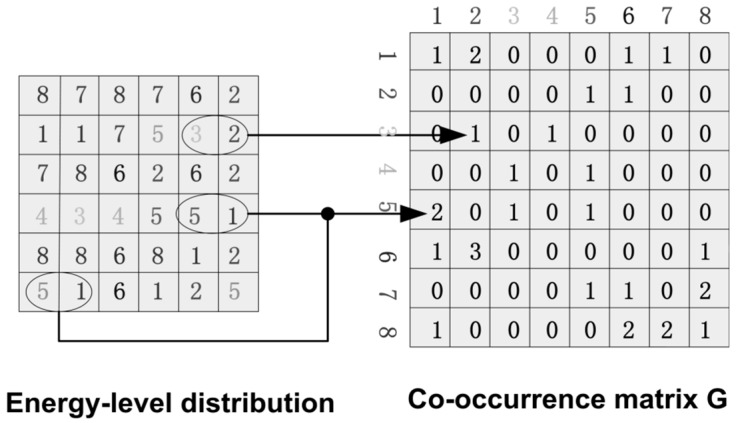
Generate a co-occurrence matrix.

Figure 5 shows the energy-level distribution histogram of motion particles when the crowd is in the normal and abnormal situations respectively. We can see that when people in a normal state, the motion particles are mostly in low level. When people get panic, a part of the motion particles transit to the high energy level. Gray level co-occurrence matrix [27] is a method commonly used to describe the gray value distribution in an image. Thus, we present the concept of energy-level co-occurrence matrix, which is used to describe the energy-level distribution. Similar to the definition of gray level co-occurrence matrix, we make Q to be an operator that defines the position of two pixels relative to each other by selecting an image of f with N possible energy-levels. Then it makes **G** to be a matrix in which the elements $g_{\mathrm{ij}}$ is the number of times that pixel pairs with energy levels $l_{i}$ and $l_{j}$ in the image of f at the position specified by Q, where 1≤i,j≤N. The matrix **G** is called energy-level co-occurrence matrix. Figure 6 shows an example of how to construct a co-occurrence matrix with N=8. A position operator Q is defined as “one pixel immediately to the right”. The array on the left is the energy-level distribution of an image, and the array on the right is the co-occurrence matrix **G**.

The presence of energy-level distribution can be detected by an appropriate position operator with the analysis of the elements in **G**. A set of useful descriptors for characterizing the contents of **G** are listed in Table 1, where *K* is the row or column of the square matrix **G**, and $p_{\mathrm{ij}}$ is the estimation of the probability for a pair of points satisfying Q, which will have values $\left(l_{i},l_{j}\right)$. It is defined as following:

(10) $$p_{\mathrm{ij}}=g_{\mathrm{ij}}/\mathrm{num}$$

where *num* is the sum of the elements of **G**. These probabilities are in the range of [0, 1], and the sum is 1.

**Figure 7:**
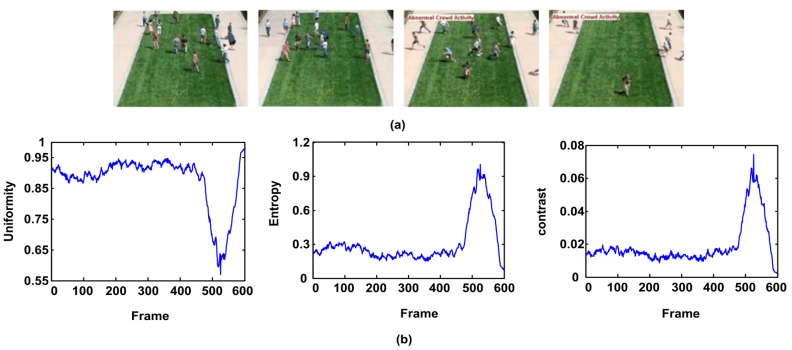
(**a**) Video Capture; (**b**) detected result of three descriptors.

In this paper, we define four position operators with distances as 1 and angles as 0°, 45°, 90° and 135° respectively. Therefore, four energy-level co-occurrence matrices can be obtained, and the value of three descriptors in Table 1 are calculated for each image respectively. Then, the mean of each descriptor is adopted to describe energy-level distribution of the motion particles. We select a sequence of the outdoor scene for the experiments, in which the people in the crowd walk aimlessly on the lawn, and then they start to run around. Figure 7a shows the sample frames of the scene. Figure 7b shows the curves of three descriptors. From the change trend of the curve we can see when people in a normal state, the particle energies are mostly in the ground state, with lower values of the entropy and contrast. Its uniformity value is higher. When crowd turns abnormal, the motion particles transit to different levels. The uniformity value drops rapidly, while the entropy and contrast value rise rapidly. We can see that the three descriptors of energy-level co-occurrence matrix can well describe the crowd state.

## 5. Experiment and Discussion 

**Figure 8:**
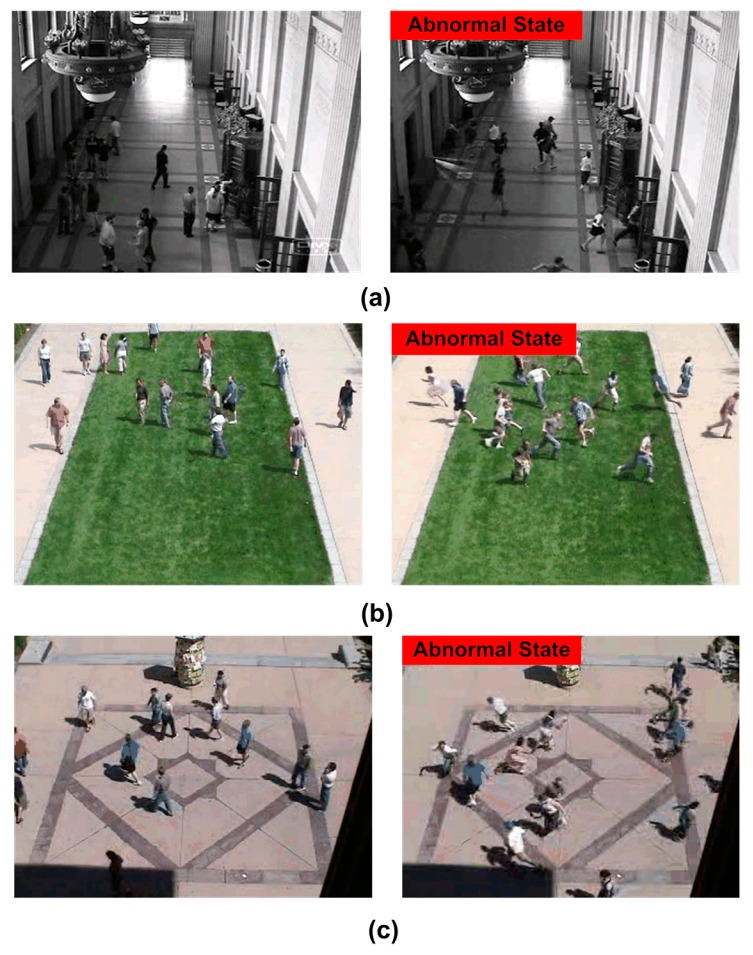
Sample frames in three different scenes of the UMN dataset. (**a**) is indoor scene; (**b**) is outdoor scene; (**c**) is outdoor square scene.

In this section, we present the experimental results of abnormal crowd behavior. The experiment is conducted on a personal computer, and the proposed system is implemented in MATLAB. The approach is tested on the publicly available dataset of the unusual crowd activity from University of Minnesota [28]. The dataset consists of 11 different videos with the escape events in 3 different indoor or outdoor scenes. Figure 8 shows the sample frames of these scenes. Each video consists of an initial part of walking as idle state and ends with sequences of running as panic state. They provide plenty of abnormal test images. The potential application of the proposed method is safety surveillance and disaster avoidance. Based on these applications, the assessment of the performance of the method pays more attention to whether it can detect the occurrence of abnormal behavior in time. Therefore, we discarded several frames of the video sequence that almost all the pedestrians escaped from their field of vision after the abnormal occurrence of a crowd. To estimate the parameters, the first 300 frames of the first clip in each scene are used for training. Table 2 shows the change rate of pedestrian area and the ground state value of each scene from the top to the bottom. 

### 5.1. Threshold Estimation

Whether the crowd is normal or abnormal is determined by comparing the three descriptor values with their thresholds. Thus, the threshold estimation is necessary. The first 300 frames of the first clip of each scene are utilized to train the parameters, and three descriptor values for every frame in the video clips are calculated accordingly. Then the threshold *T* for different descriptors in different scenes is estimated by the following formula [29]:

(11) $$\begin{array}{c} {}[T{]}_{V_{s}}=\arg\;\underset{i=1\ldots 300}{\max}{\left[{\mathrm{feature}}_{m}\right]}_{i}+\\ \arg\;\underset{i=1\ldots 300}{\min}{\left[\frac{1}{{\left(2\pi \right)}^{2}}\sum_{j=0}^{\infty }\frac{{\left(-1\right)}^{j}{\left({\mathrm{feature}}_{m}\right)}^{2j+1}}{j!\left(2j+1\right)}\right]}_{i} \end{array}$$

where *V_S_* is the sample video for different scenes (where *s* = 1, 2, 3), *i* is the number of frames in the video *V_S_* *(where i* = 1, 2, …, 300), and *feature_m_* is the value of the *m*th descriptor (where *m* = 1, 2, 3). To well estimate the threshold, we add some minimum Gaussian errors as margin on the base of the maximum of descriptor. When estimating the threshold of uniformity descriptor, we inverse the test result of each frame, and then flip it again after the threshold value is acquired. Table 3 lists the threshold values of three descriptors in different scenes.

### 5.2. The Results of Abnormal Crowd Behavior Detection Using Different Features 

In this paper, we use three descriptors extracted from energy-level co-occurrence matrix to describe the crowd state. In order to evaluate the performance of the three descriptors, several crowd videos in the UMN dataset are used in this experiment. The results show that the proposed three features can distinguish the normal or abnormal crowd behavior. We choose one video clip from each scene, and list the test results. The results report that the proposed method can be used to detect the abnormal crowd behavior. To avoid noise, the system alarm [30] is arisen only when the value is greater or lower than its threshold in 10 consecutive frames. The test results are shown as follows.

**Figure 9:**
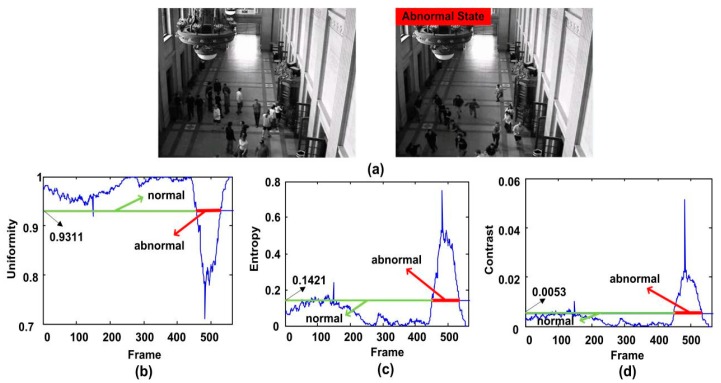
The qualitative results of the abnormal behavior detection for the third clip of the first scene of UMN dataset. (**a**) shows two normal and abnormal frames, (**b**–**d**) show the uniformity, entropy and contrast features respectively.

First of all, we select the fourth clip of the indoor scene in the UMN dataset to show the performance of the proposed feature. In this clip, people are walking freely or standing for chatting. Then they start running in one direction. Our algorithm can detect the anomaly in time. Figure 9b shows that the value of uniformity operator is lower than the threshold for more than 10 consecutive frames starting from the 456th frame. The alarm is arisen at the 466th frame when the crowd anomaly is detected. It also can be seen that a sharp curve occurs at the 149th frame due to the actually noise. However, it only lasts one frame, and our system avoids false alarm successfully. When using entropy operator for the experiment, its value is higher as 0.1421 for 10 consecutive frames, and the system alarm is arisen. As shown in Figure 9c, we can see a sharp peak after the 451st frame. So after 10 frames, namely at the 461st frame, the alarm is triggered. Meanwhile, test results show that the value is greater than the threshold for several frames starting from the 82th frame to the 120th frame. Since they are no more than 10 frames, alarms are not triggered in this case. From Figure 9d, we can see a sharp peak after the 451st frame. By using contrast operator for testing, the abnormal can also be detected. However, there are 13 consecutive values greater than their thresholds starting from the 120th frame, and the false alarm occurs. We need to give an all clear manually, and trigger the alarm at the 461th frame.

**Figure 10:**
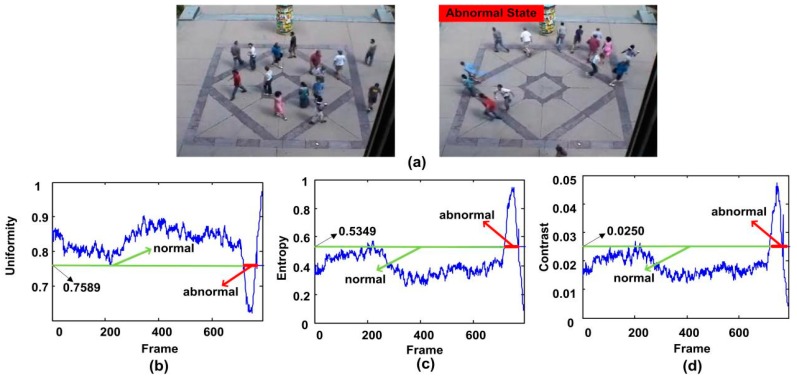
The qualitative results of the abnormal behavior detection for the third clip of the second scene of UMN dataset. (**a**) shows two normal and abnormal frames, (**b**–**d**) show the uniformity, entropy and contrast features respectively.

The second clip shows that people walk freely in a low speed, and then start to run in different directions. It is the third clip in the outdoor square scene of the UMN dataset. From Figure 10b to Figure 10d, it can be seen that all of the three descriptors are suitable for detecting the crowd abnormal behavior. When using uniformity operator for testing, it can trigger the alarm at the 727th frame to prompt the abnormal events, without false alarm. Figure 10c,d present the curve of entropy operator and contrast operator respectively. They all trigger the alarm at the 728th frame. Meanwhile we can see our method avoids the false alarm successfully at the 202th frame and 217th frame respectively.

**Figure 11:**
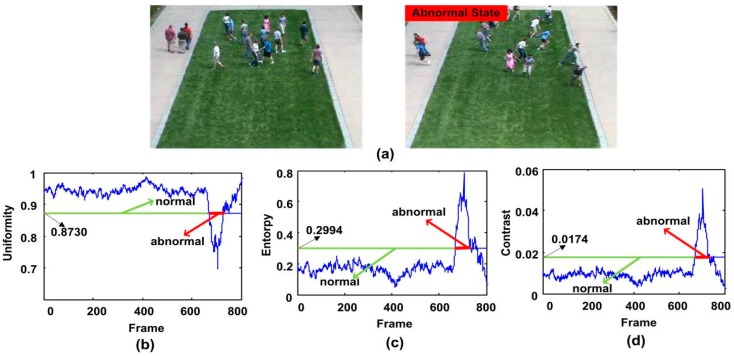
The qualitative results of the abnormal behavior detection for the second clip of the third scene of UMN dataset. (**a**) shows two normal and abnormal frames; (**b**–**d**) show the uniformity, entropy and contrast features respectively.

The third experiment sequence is the second clip of the outdoor lawn scene of UMN dataset, in which people walk freely on the grass, and then start to run in different directions. The test results of three descriptors are shown in Figure 11. It can be seen that the detection results of three descriptors are very well. The alarm is triggered at the 679th, 680th and 680th frame respectively.

### 5.3. Integrating the Proposed Three Features 

From the experiments in Section 5.2 we can find that there is false alarm using a single descriptor to detect the crowd behavior. Therefore, in this paper, we use three descriptors for testing at the same time to eliminate the error. The crowd behavior abnormal is detected only if all of the three descriptors judge it as a crowd abnormal behavior.

**Figure 12:**
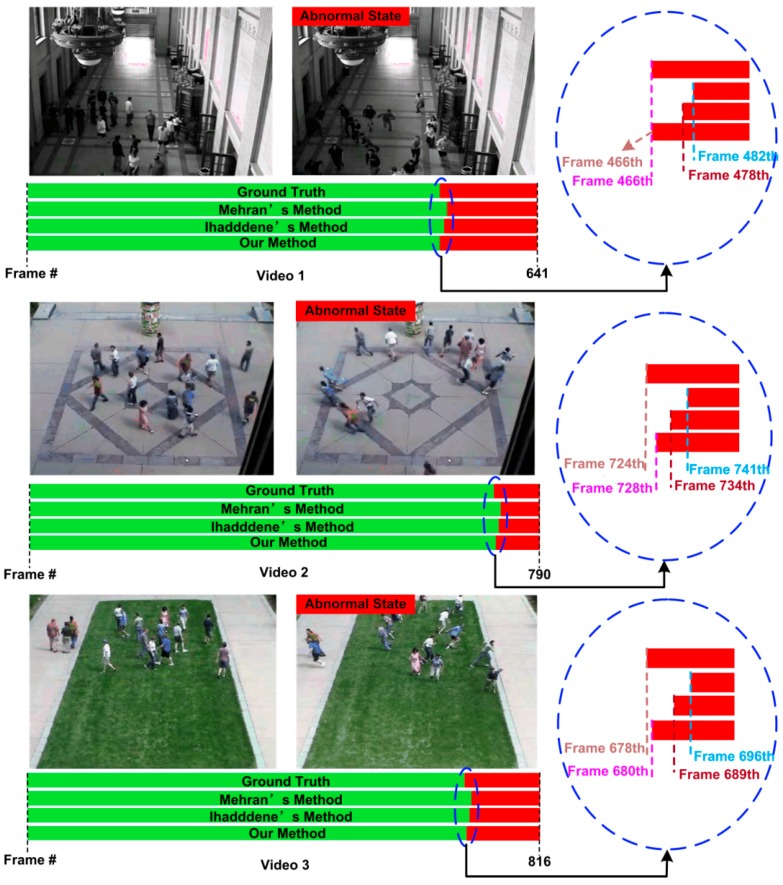
Comparison of the use of the proposed method with other classical methods for detection of the abnormal behaviors in the UMN dataset.

In order to justify the superiority of this method, we compare our model with the ones from Ihaddadene et al. [31] and Mehran et al. [18] using the three sequences the same as the experiments in Section 5.2. Figure 12 shows some of the qualitative results for the detection of abnormal scenes. The bar represents the label of each frame in that video. The green color represents the normal frames and red as abnormal frames. It can be seen that our method can alarm to remind earlier than other two classical methods. For the first scene, when an abnormal event occurs, our method begins to trigger the alarm in a timely manner, while the methods from reference [18,31] detect abnormal after 16 and 12 frames respectively. For the second scene, our method trigger the alarm when the crowd become abnormal after four frames, which is earlier than the testing results of the method in the reference [18,31] with 13 and six frames respectively. For the third scene, our method has two frames later than the ground truth to trigger the alarm when the crowd begins to run. It is still earlier than the testing results of the methods from reference [18,31] with 16 and nine frames respectively.

### 5.4. Discussion 

The advantage of the proposed method is that it is insensitive to the change of point of view. The qualities of different particles in a video frame are distributed as different mass according to the distance between the pedestrian and the camera. With this method, the camera perspective effect can be reduced. Another advantage is the feature of the energy level. Different with energy and velocity, the energy level model is more suitable for descripting the panic motion of a crowd. However, the proposed method is sensitive for the threshold estimation and the number of pedestrians in the scene. If the threshold is set as a small value, some false alarm will occur in normal situation. On the contrary, if we set a larger threshold, the timeliness of the alarm will be affected. Thus, in practical applications, the threshold should be more carefully selected according to the specific scene. However, the number change of pedestrians will influence the detection result. For example, if almost every pedestrian run out of the surveillance scene, the proposed method maybe treat it as a normal status. Fortunately, in real applications for safety surveillance and alarm, to detect and alarm the abnormal behavior in time is the most important thing.

The proposed method has potentials and some applicability in other crowd analysis methods. The proposed method is very easy to be integrated into other different methods. Firstly, in many physical models (such as force and energy), the mass is set as 1, which is not a good solution. Combining the proposed mass estimation method with other physical based models can achieve better results of crowd abnormal detection. Secondly, the energy level model proposed for crowd abnormal behavior detection is also suitable for integrating into other method such as entropy and probability model. Finally, modeling and simulation of crowd motion [32,33,34,35,36,37] is an important research topic in the field of population disaster avoidance. The proposed energy level model is also suitable for the application of crowd modeling and simulation.

## 6. Conclusions 

We propose an effective approach to detect crowd abnormal behavior in video streams using the change of energy-level distribution. This method has two main advantages. We assign different numbers of particles that have different distance from the camera using flow field visualization based method to reduce the influence brought by the camera perspective effect. However, we build a particle kinetic model and present a concept of energy-level co-occurrence matrix. Then, the energy-level distribution of particle is described with co-occurrence matrix descriptors. The status of the crowd is determined by analyzing the change trend of the descriptor values. The results indicate that our method is effective in detecting the abnormal crowd behaviors. 

In future work, we plan to design more effective physical models to describe crowd behavior at the macro and micro level. Recently, convolutional neural network (CNN) has shown excellent performance in feature extraction. However, deep learning related methods usually require a large number of sample data for training. For crowd panic and disastrous events detection, it is very hard to collect enough data because the disaster cannot be reproduced many times. In future work, we will try to collect as much images and video data as possible from the Internet, and will consider using deep learning to resolve the problem of crowd abnormal behavior detection. 

## Figures and Tables

**Table 1:** Descriptors used for characterizing co-occurrence matrix.

Descriptor	Explanation	Formula
Uniformity	A measure of uniformity in the range [0, 1]. Uniformity is 1 for a constant energy-level.	$\overset{K}{\underset{i=1}{\sum }}\overset{K}{\underset{j=1}{\sum }}p_{\mathrm{ij}}^{2}$
Entropy	Measures the randomness of the elements of G.	$-\sum_{i=1}^{K}\sum_{j=1}^{K}p_{ij}\log_{2}p_{ij}$
Contrast	A measure of energy-level contrast between a particle and its neighbor over the entire image.	$\overset{K}{\underset{i=1}{\sum }}\overset{K}{\underset{j=1}{\sum }}{\left(i-j\right)}^{2}p_{\mathrm{ij}}$

**Table 2:** The parameter values under different scene.

	Scene 1	Scene 2	Scene 3
*k*	0.412	0.628	0.680
*E*_ground_	0.490	0.692	0.849

**Table 3:** The threshold of three descriptors in different scene.

	Scene1	Scene 2	Scene 3
Uniformity	0.9311	0.7589	0.8730
Entropy	0.1421	0.5349	0.2994
Contrast	0.0053	0.0250	0.0174
